# Near-absolute polarization insensitivity in graphene based ultra-narrowband perfect visible light absorber

**DOI:** 10.1038/s41598-018-33609-2

**Published:** 2018-10-12

**Authors:** Deniz Umut Yildirim, Amir Ghobadi, Ekmel Ozbay

**Affiliations:** 1 NANOTAM-Nanotechnology Research Center, Bilkent University, 06800 Ankara, Turkey; 2 Department of Electrical and Electronics Engineering, Bilkent University, 06800 Ankara, Turkey; 3 Department of Physics, Bilkent University, 06800 Ankara, Turkey; 4 UNAM-Institute of Materials Science and Nanotechnology, Bilkent University, Ankara, Turkey

## Abstract

Strong light-graphene interaction is essential for the integration of graphene to nanophotonic and optoelectronic devices. The plasmonic response of graphene in terahertz and mid-infrared regions enhances this interaction, and other resonance mechanisms can be adopted in near-infrared and visible ranges to achieve perfect light absorption. However, obtaining near-absolute polarization insensitivity with ultra-narrow absorption bandwidth in the visible and near-infrared regimes remains a challenge. In this regard, we numerically propose a graphene perfect absorber, utilizing the excitation of guided-modes of a dielectric slab waveguide by a novel sub-wavelength dielectric grating structure. When the guided-mode resonance is critically coupled to the graphene, we obtain perfect absorption with an ultra-narrow bandwidth (full-width at half-maximum) of 0.8 nm. The proposed design not only preserves the spectral position of the resonance, but also maintains >98% absorption at all polarization angles. The spectral position of the resonance can be tuned as much as 400 nm in visible and near-infrared regimes by tailoring geometrical parameters. The proposed device has great potential in efficient, tunable, ultra-sensitive, compact and easy-to-fabricate advanced photodetectors and color filters.

## Introduction

Ever since the experimental isolation of graphene in 2004^1^, this monolayer of carbon atoms in a honeycomb lattice has attracted enormous attention because of its unique electronic, optical, and mechanical properties^2–4^, and paved the way for the advent of other two-dimensional materials^5,6^. The ultra-wideband spectral response and the huge carrier mobilities make graphene a strong candidate as a backbone of novel nanophotonic and optoelectronic devices. Indeed, graphene-based solar cells^7,8^, saturable absorbers for the mode-locked ultra-fast lasers^9–11^, photodetectors^12,13^, transparent electrodes^14–16^, optical modulators^17,18^ and third harmonic generation^19,20^ are demonstrated. Further incorporation of graphene into high-performance optical devices makes strong light-graphene interaction compulsory. Localized surface plasmons and surface plasmon polaritons supported by graphene in the terahertz (THz) and mid-infrared (MIR) regimes can enhance this interaction and perfect absorption can be achieved^21–25^. In contrast, in the visible and near-infrared range (Vis-NIR), graphene’s optical response is dominated by interband transitions, giving a real conductivity with no plasmonic response and graphene effectively mimics a dielectric with considerable loss^26^. Unpatterned and suspended graphene absorbs *A* = *πα* = 2.3% of the normally incident light without spectral selectivity, defined by the fine structure constant *α* = *e*^2^/ℏ*c*. *A* = 2.3% is actually a significant value for a one-atom thick material. Nevertheless, the absolute strength of the absorption is low and it limits graphene’s further application in optoelectronics and photovoltaics in the visible spectrum, an example related to our work being low responsivity in photodetectors^27,28^.

Since graphene plasmons cannot be excited outside the THz and MIR ranges, graphene has to be coupled to other resonant mechanisms to improve the light absorption in the Vis-NIR ranges. Enormous research efforts have been devoted to enhancing the local optical field around graphene and increase light-graphene interaction in the Vis-NIR ranges. The methods to achieve these can be classified into four categories. First, graphene can be placed inside a Fabry-Pérot (FP) cavity^29,30^. This method can confine large optical fields and significantly enhance the absorption in graphene. However, these designs require deposition of a large number of dielectric layers (25 pairs of AlAs and AlGaAs for the bottom reflector and 7 pairs of SiO_2_ and Si_3_N_4_ layers for top reflectors, reported in ref.^29^) on top of graphene. This results in not only fabrication difficulty, but also bulky devices. Another option is placing graphene near plasmonic nanostructures^31–34^ and relying on the near-field enhancement effect of surface plasmons or surface plasmon polaritons. This method suffers from absorption peak broadening because of the background absorption coming from the metals, which is detrimental to color sensitivity. Moreover, significant Joule losses decrease device efficiencies^35^.

The third and fourth methods can be classified together as coupling of graphene to guided-mode resonances (GMRs) that define the phase-matched coupling between the free-space radiation and the supported (guided) modes with a leaky nature due to their complex wavenumber^36–42^. The difference between two methods is the structures where GMRs are excited. The third method is to couple graphene to the GMRs of photonic crystal slabs (PhCs)^43^ or 1-dimensional photonic crystals (1DPCs), excited by sub-wavelength grating (SWG) couplers^44,45^. The guided-mode leaks out evanescent waves to graphene. Critical coupling of the guided-modes to graphene then produces perfect absorption. Notably, the latter can achieve an ultra-narrow absorption bandwidth of 0.03 nm due to placing graphene far away from 1DPC^45^. Although promising in theory, this method enforces extreme fabrication accuracy on not only the SWG, but also on the 1DPCs to achieve the target wavelength and store very high energy in 1DPC to achieve critical coupling. Similar to the methods that adopt FP cavity, this method also results in bulky components. The final method is to excite the GMRs of either unmodulated slab-waveguide structures beneath the grating^46–50^, or the grating itself  ^51^, which acts as a waveguide whose core is refractive-index modulated bulk^37,38^. These GMRs then interact with graphene to increase light-graphene interaction. Compared to the third method, this method offers more compact designs and a more facile fabrication process. This is because it does not require 1DPC, but only a SWG, two or three dielectric films with only one being patterned, and possibly a perfect back metal mirror or Bragg reflector. However, the downside is the larger bandwidth because low-Q resonances can be coupled to graphene, due to the smaller distance between GMR and graphene. Thus, there is a fundamental trade-off between the two methods in terms of the fabrication complexity and Q-factor of resonances. While the previous studies for the guided-mode resonances focused mainly on the absorption enhancement, polarization insensitivity remained an issue, because of incorporating 1-dimensional SWGs. Although polarization-insensitivity between s and p-polarizations is achieved by the square lattice in ref.^43^, the bandwidth, measured as full-width at half-maximum (FWHM), 14 nm is quite high because of the circular shape of the holes. The sharp features of gratings allow for enhanced field localizations and high-Q resonances. Hence, an ideal unit-cell would use a rectangular grating structure with rotational symmetry to preserve both strong light absorption and achieve polarization insensitivity.

In the present paper, we employ the finite-difference-time-domain (FDTD) method to numerically propose a novel design that uses a 2-dimensional sub-wavelength grating structure to excite the GMRs of an unmodulated slab waveguide. The critical coupling of the GMRs to graphene yields 99.2% light absorption with 0.8 nm FWHM at 678 nm, under p-polarized (TM) light. The proposed device preserves perfect absorption >98% at all source polarizations as well as the spectral position of the resonance. To the best of our knowledge, this is the first study that illustrates an FWHM below 1 nm with near-absolute polarization independence. Furthermore, the presence of a waveguide structure, instead of 1DPCs or FP cavities, relaxes the device fabrication. The spectral position of the resonance is easily tunable by changing the key geometrical parameters of the device. We first show the enhancement of graphene absorption in a simple optimized grating-core-substrate structure and scrutinize the effect of geometrical parameters on the device characteristics. In the second part, we add a metal back mirror and optimize the geometry accordingly to achieve critical coupling, and hence perfect absorption with the ultra-narrow bandwidth. We discuss the design considerations to maximize absorption strength and minimize absorption bandwidth, for each case.

## Results and Discussion

### Exciting guided-mode resonances of a slab-waveguide to improve light-graphene interaction

**Figure 1:**
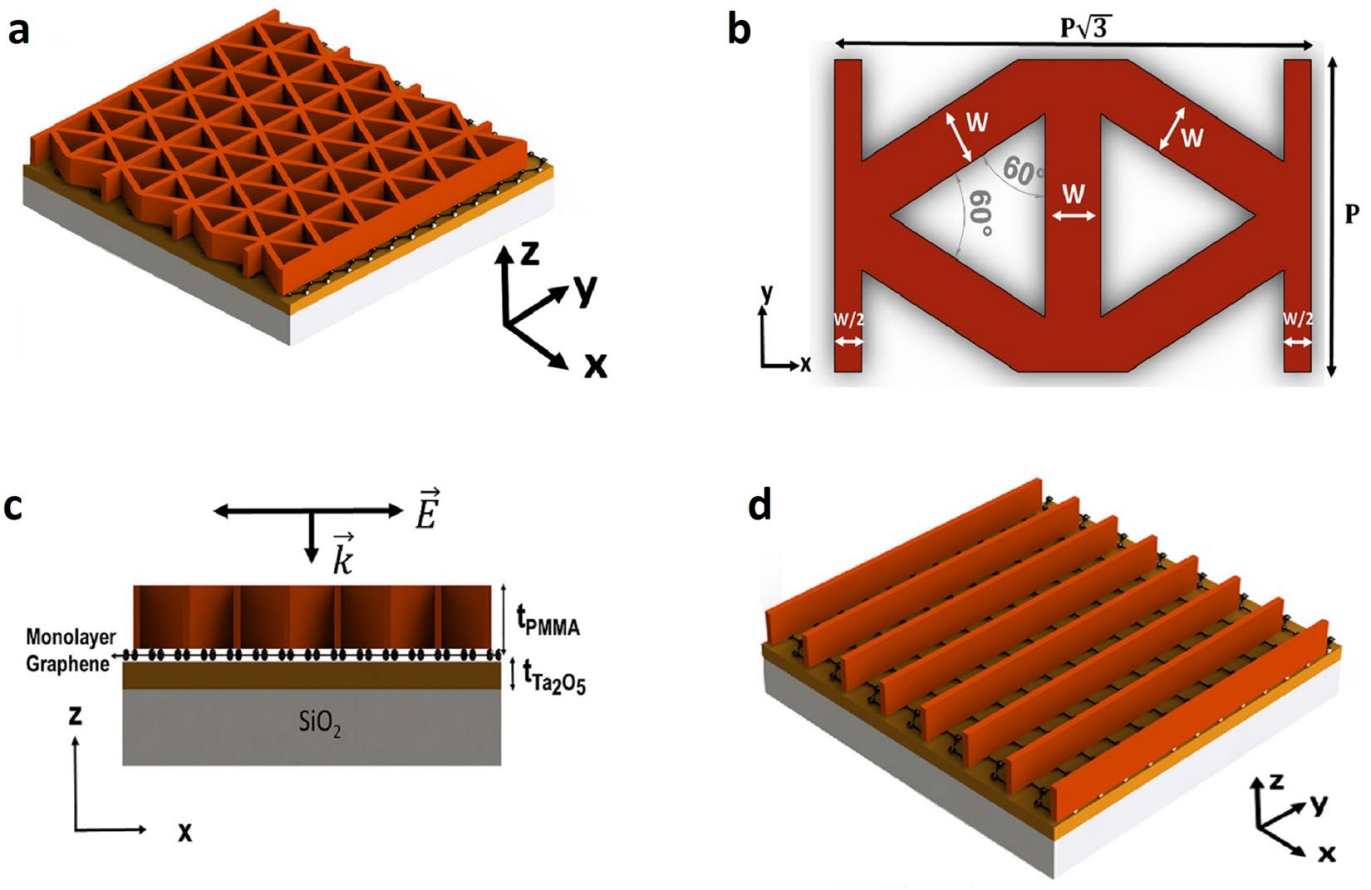
The proposed design, its unit-cell and the structure with the classical 1D grating. (**a**) Device I to excite GMRs and enhance light-graphene interaction. (**b**) Unit-cell of Device I, detailing the 2D grating, seen from top-view. (**c**) Dielectric layers and the monolayer graphene of Device I, seen from side-view. (**d**) The structure with the commonly used 1D-grating. In (**c**), the device is illuminated by p-polarized plane wave.

Our proposed device in Fig. 1a is designed to excite the guided-modes in the high-refractive index Tantalum pentoxide (Ta_2_O_5_), unmodulated core dielectric region by a unique 2-dimensional sub-wavelength polymethyl-methacrylate (PMMA) dielectric grating. The structure is supported by glass (SiO_2_) substrate. We call this two-port structure Device I. Figure 1b shows the top-view of Device I to indicate the details of our novel 2D sub-wavelength grating, which is based on our previous effort on achieving polarization-insensitivity in wideband plasmonic absorbers^52^. Another equivalent unit-cell for the structure is presented in Fig. S1. The dielectric layers and monolayer graphene of Device I are shown in Fig. 1c. The critical design parameters in determining the spectral position of GMRs and the absorption strength are the thickness of the core region, ${{\rm{t}}}_{{{\rm{Ta}}}_{{\rm{2}}}{{\rm{O}}}_{{\rm{5}}}}$; the periodicity, P, of the unit cell in y-direction, the grating width w, and the grating thickness t_PMMA_.

To investigate the spectral response of Device I, we utilize finite-difference-time-domain simulations. In the numerical simulations, we illuminated the unit-cell with a broadband plane-wave at normal incidence whose electric-field is polarized in x-direction (*ϕ* = 0, p-polarization). In the x and y directions, we use periodic boundary conditions, while in the z-direction perfectly matched layers (PML) are adopted and they are placed 5 wavelengths away from the structure. We use two monitors that are at an adequate distance away from the structure to find the reflected (R) and transmitted (T) power. Absorption is then found by using *A* = 1 − *R* − *T* formula. The dispersion effect of the dielectric materials are retrieved by ellipsometric measurements, and they are in good agreement with the models based on Sellmeier equation^53^. In the material fits, the only non-zero extinction coefficient comes from Ta_2_O_5_, and is equal to 10^−5^. It is then reasonable to regard the device as lossless without graphene. Graphene is modeled as a 2-dimensional material with infinitesimal thickness and a complex surface conductivity that includes the chemical potential, scattering rate and temperature effects, based on Kubo formulation^54^. The surface conductivity is divided into intraband and interband contributions, as given in Eqns 1 and 2, respectively.

1 $$\begin{array}{rcl}{\sigma }_{g}(w,{\mu }_{c},{\rm{\Gamma }},T) & = & {\sigma }_{g}^{intra}(w,{\mu }_{c},{\rm{\Gamma }},T)+{\sigma }_{g}^{inter}(w,{\mu }_{c},{\rm{\Gamma }},T)\\ {\sigma }_{g}^{intra}(w,{\mu }_{c},{\rm{\Gamma }},T) & = & \frac{j{e}^{2}}{\pi {\hslash }^{2}(w-j2{\rm{\Gamma }})}{\int }_{0}^{\infty }\,\varepsilon (\frac{\partial {f}_{d}(\varepsilon )}{\partial \varepsilon }-\frac{\partial {f}_{d}(\,-\,\varepsilon )}{\partial \varepsilon })d\varepsilon \end{array}$$

2 $${\sigma }_{g}^{inter}(w,{\mu }_{c},{\rm{\Gamma }},T)=\frac{-\,j{e}^{2}(w-j2{\rm{\Gamma }})}{\pi {\hslash }^{2}}{\int }_{0}^{{\rm{\infty }}}\,(\frac{{f}_{d}(\,-\,\varepsilon )-{f}_{d}(\varepsilon )}{{(w-j2{\rm{\Gamma }})}^{2}-4(\varepsilon /\hslash {)}^{2}})d\varepsilon $$

where *j* is the imaginary unit, *e* is the elemental charge, *w* is the angular frequency, *T* is the temperature, *ℏ* = *h*/2*π* is the reduced Planck’s constant, Γ is the scattering rate, and *μ*_*c*_ is the chemical potential. ${f}_{d}(\varepsilon )={({e}^{(\varepsilon -{\mu }_{c})/{k}_{b}T}+\mathrm{1)}}^{-1}$ is the Fermi-Dirac distribution and *k*_*b*_ is Boltzmann’s constant.

**Figure 2:**
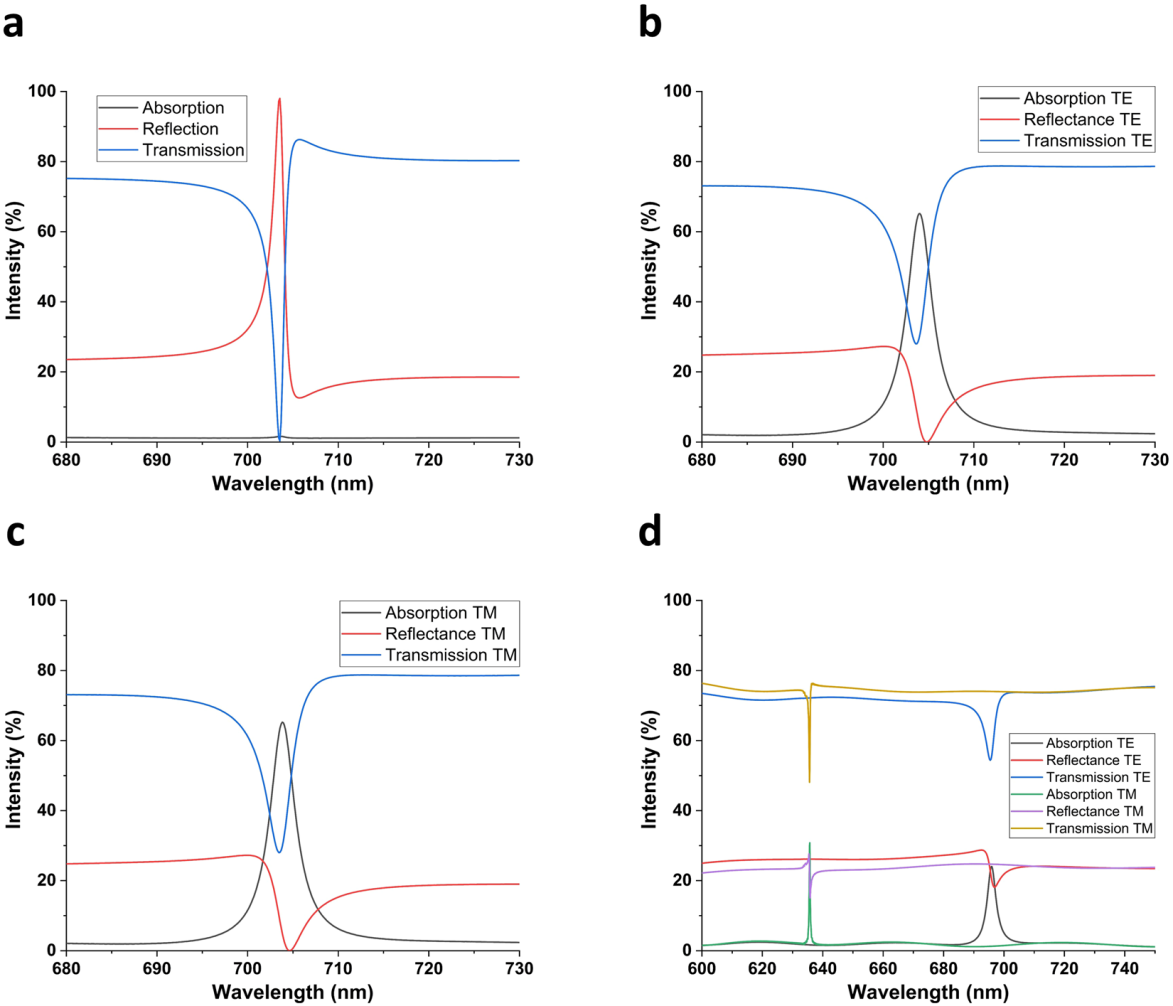
Excitation of GMRs and enhancement of light-graphene interaction. Transmission, Reflectance and Absorption spectra for the proposed device (**a**) without graphene, (**b**) with graphene, under s-polarized (TE) light; (**c**) with graphene, under p-polarized (TM) light; (**d**) with 1D grating and graphene, under TE and TM polarized light to show polarization dependency.

First of all, we set the design parameters ${{\rm{t}}}_{{{\rm{Ta}}}_{{\rm{2}}}{{\rm{O}}}_{{\rm{5}}}}$, P, w, and t_PMMA_ to their optimum values of 90 nm, 500 nm, 100 nm (0.2P) and 220 nm, respectively. We simulated Device I without graphene to gain insight about the resonance excitation and to confirm that the device is almost lossless. The simulation result in Fig. 2a shows a sharp guided-mode resonance peak at 703.565 nm, and only 1% absorption due to Ta_2_O_5_. It is then safe to associate the absorption in subsequent simulations to graphene. In Fig. 2a, when the device is off-resonant, no diffracted order is coupled to the waveguide, so the device essentially acts as a multilayer film that mostly transmits the incoming wave, and some part is reflected due to Fresnel reflection. When on-resonant, however, one of the diffracted orders couple to the guided-wave in slab waveguide core. The initially diffracted waves of incident wave and the re-diffracted waves of the guided-wave then interfere destructively to produce a transmission filtering response. Essentially, the condition for the particular diffracted order to be guided and the other diffracted orders interfering destructively lead to the same condition^36^. Another condition for the occurrence of the resonance is the phase-matching condition, i.e. the real part of the complex wavevector component in the travelling direction of the guided-mode is equal to the components coming from the reciprocal lattice vector of grating and the incident wave^21,36,47,48^. Eq. 3 provides the phase-matching condition for the devices with 1D grating, as in Fig. 1d under normally incident light. We call the device in Fig. 1d Device 0.

3 $$\begin{array}{rcl}\beta ={k}_{0}{n}_{eff} & = & m\frac{2\pi }{{\rm{\Lambda }}}\\ {n}_{eff} & = & m\frac{{\lambda }_{0}}{{\rm{\Lambda }}}\end{array}$$

where *β* is the propagation wavenumber along the guiding direction, *k*_0_ is the free-space wavenumber, *n*_*eff*_ is the effective refractive index of the mode inside the core, m is the diffraction order, λ_0_ is the free-space wavelength and Λ is the grating period. Writing an analytical expression for the phase-matching condition in Device I is cumbersome because our grating diffracts incoming waves in not only the x-direction but also in the y-direction, as opposed to the explicitly used devices with 1D grating^44–51^ does. Therefore, the guided-mode’s propagation direction is not simply the x-direction as in Device 0. This makes numerical methods necessary to fully discern the resonances. Another interesting feature of GMRs is their Fano lineshape. It arises due to the interaction between a sharp resonance channel and the broad radiation continuum^55^. Critically, the zeroth order diffracted wave is a propagating wave, while all other diffracted orders are evanescent due to the sub-wavelength nature of the grating. Therefore, we have evanescent coupling of the incident wave to the guided-mode.

We then sandwiched monolayer graphene between the grating and Ta_2_O_5_ to increase the light-graphene interaction, thanks to the guided-wave confined in the waveguide^28^. The spectral response of the configuration is shown in Fig. 2b,c for the s (plane of incidence is XZ plane, E-field polarized in y-direction) and p (plane of incidence is XZ plane, E-field polarized in x-direction) polarized incident waves, respectively. From these figures, it is obvious that the structure gives almost identical results in terms of both the absorption strength and the resonance’s spectral position (65.22% at 703.82 nm for p, 65.17% at 705.05 nm for s). The FWHM of the resonance is also maintained at 3.5 nm. To demonstrate the polarization insensitivity of Device I compared to Device 0, we examined the spectral response of the latter with the structural parameters of the former under s and p-polarized lights. Figure 2d shows the absorption spectrum for Device 0. Although Device 0 can be optimized to maximize absorption strength, the key point in Fig. 2d is that both the spectral position and the strength of absorption are affected from incident light’s polarization. Ref.^47^ also found that simple 1D grating and unmodulated slab waveguide structure has a theoretical maximum absorption of 60% without the addition on back-mirror. We, therefore, attribute both the near-stable spectral position of resonance and slightly enhanced absorption to our novel grating design.

Figure 2b,c demonstrate that absorption at the resonance arises mostly due to the detriment of reflection, while a considerable amount of transmission still remains, in harmony with the results reported in ref.^47^. While Fig. 2a for Device I without graphene shows almost the complete suppression of transmission, in Device I with graphene it can never be zero because of two reasons. First, the guided-wave itself is leaky and leaks out evanescent waves to not only graphene (which is the reason of absorption enhancement) but also to the substrate. More importantly, the re-diffracted waves of the guided-mode cannot fully cancel out the initially diffracted waves because of their decreasing amplitude due to graphene.

**Figure 3:**
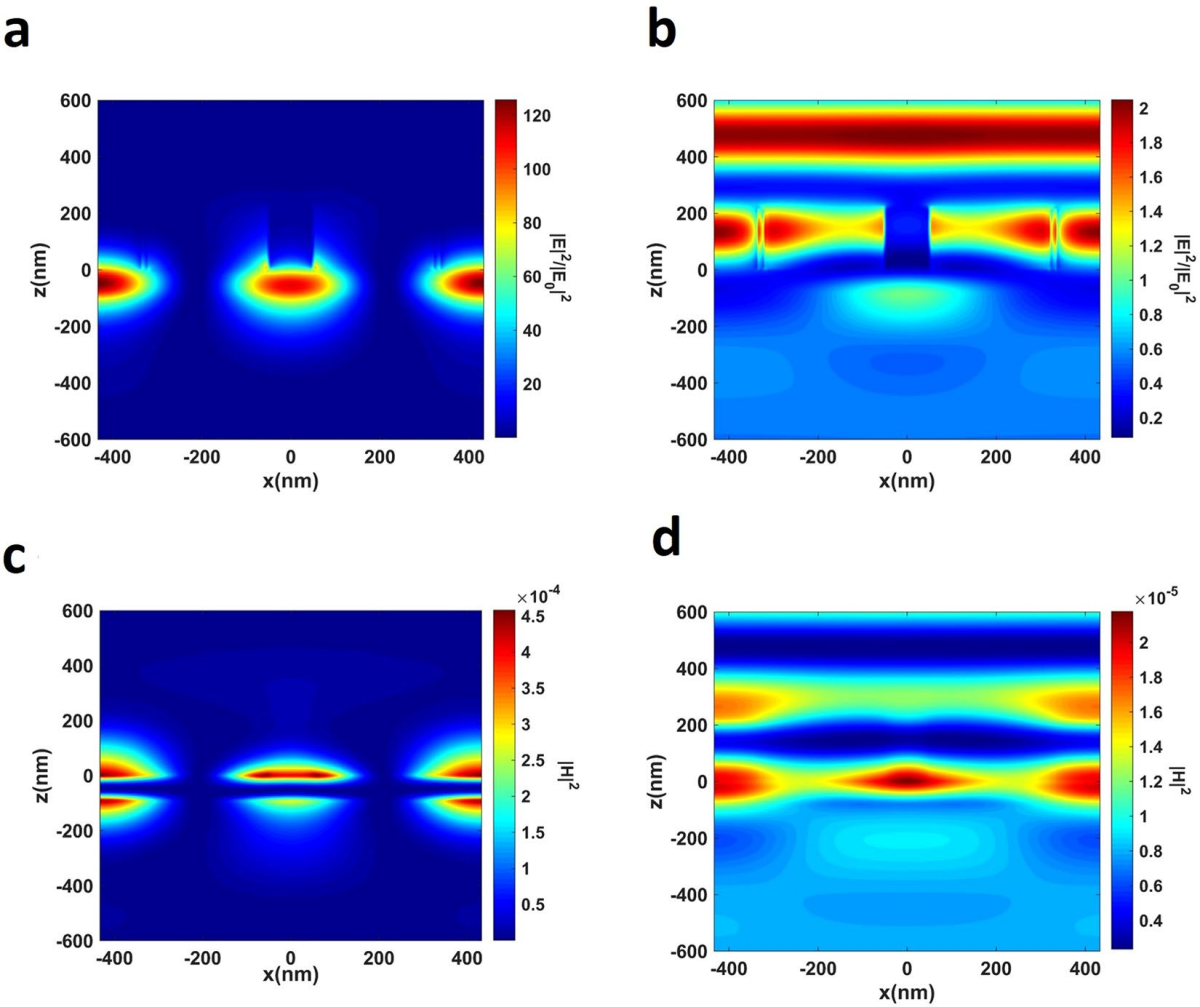
Distribution of electric and magnetic field in the on and off-resonant conditions. |E|^2^/|E0|^2^ at XZ plane during **(a)** on-resonance at 703.82 nm; **(b)** off-resonance at 751.9 nm; |H|^2^ at XZ plane during **(c)** on-resonance, **(d)** off-resonance. |E|^2^ and |H|^2^ are the modulus of electric and magnetic field inside the device, |E0|^2^ denotes that of incident light. Graphene is located at z = 0.

To get further comprehension about the improvement in the light-graphene interaction, we extracted the electric and magnetic field profiles for both off-resonant and on-resonant cases. A comparison of the fields in the off and on-resonant cases, shown in Fig. 3, clearly indicates a strong field enhancement and localization on graphene. While |E|^2^ is enhanced two orders of magnitude with respect to the incident light, |H|^2^ is enhanced one order of magnitude. The enhanced optical field on graphene is directly responsible for the absorption enhancement, in accordance with Eq. 4^56,57^,

4 $$A(\lambda )=\frac{\int {\int }_{x,y\in layer}\,w{\varepsilon }_{0}n^{\prime\prime} n^{\prime} |E{|}^{2}dxdy}{I(\lambda )}$$

where *ε*_0_ is the vacuum permittivity, *λ* and *I*(*λ*) are the wavelength and the intensity of the light, respectively, *n*″ and *n*′ denote the imaginary part (extinction coefficient) and real part of the complex refractive index, respectively. The enhancement of fields is slightly higher than the designs incorporating slab waveguide as the guiding layer^46–50^. We also observe that the standing wave pattern is created in both the z and x-directions. While the former is because of the waveguiding effect, the latter is due to the refractive index modulation in the x-direction^45^. The electric field profile in Fig. 3a implies that the first order mode of the slab waveguide is excited. This is entirely expected because of the deep sub-wavelength thickness of the core region, as the excitation of higher order modes of a waveguide requires larger core thickness^58,59^.

#### Reasoning of our 2D SWG to achieve polarization insensitivity

Because it is the most important aspect of our design, we devoted this subsection to the further dissection of polarization insensitivity in Device I, by analyzing what causes the devices with 1D grating, such as Device 0, to be polarization sensitive in the first place. Firstly, different boundary conditions imposed on different polarization states, which results in form birefringence^60^. In addition, when the guided-mode travels along the periodically segmented waveguide^61^, the periodicity of the grating defines Bloch modes. Different effective refractive indices of the Bloch modes in the grating region^42^ consequently define different effective mode refractive indices for TE and TM modes of the waveguide. The excitation condition of TE and TM modes are different on top of this^58,59,62^. Thus, the devices utilizing 1D gratings show polarization dependency^42^ as they excite the GMRs at different wavelengths. Moreover, dissimilar field distributions result in different absorption strengths.

Without considering the waveguiding effects and the complicated properties of Bloch modes, we adopted Rytov’s formulas given in Eqns 5 and 6 for s and p-polarized waves as our guideline in achieving polarization independence. Rytov’s formulas use different boundary conditions forced on different polarization states to find the effective permittivity of the medium to the zeroth order, for deep-subwavelength 1D gratings with sufficient thickness^63^.

5 $${n}_{\parallel }^{2}=f{n}_{1}^{2}+\mathrm{(1}-f){n}_{2}^{2}$$

6 $$\frac{1}{{n}_{\perp }^{2}}=f\frac{1}{{n}_{1}^{2}}+\mathrm{(1}-f)\frac{1}{{n}_{2}^{2}}$$

where *f* is the fill-factor of grating, *n*_1_ and *n*_2_ are the refractive indices of the grating material, and its surrounding, respectively. ${n}_{\parallel }$ and *n*_⊥_ are the refractive indices of the effective medium for the s-polarized (E-field parallel to grating grooves), and p-polarized waves (E-field perpendicular to grating grooves), respectively. Any arbitrary incident polarization can then be decomposed into a superposition of s and p-polarizations to find the effective refractive index as a weighted sum of ${n}_{\parallel }$ and *n*_⊥_.

**Figure 4:**
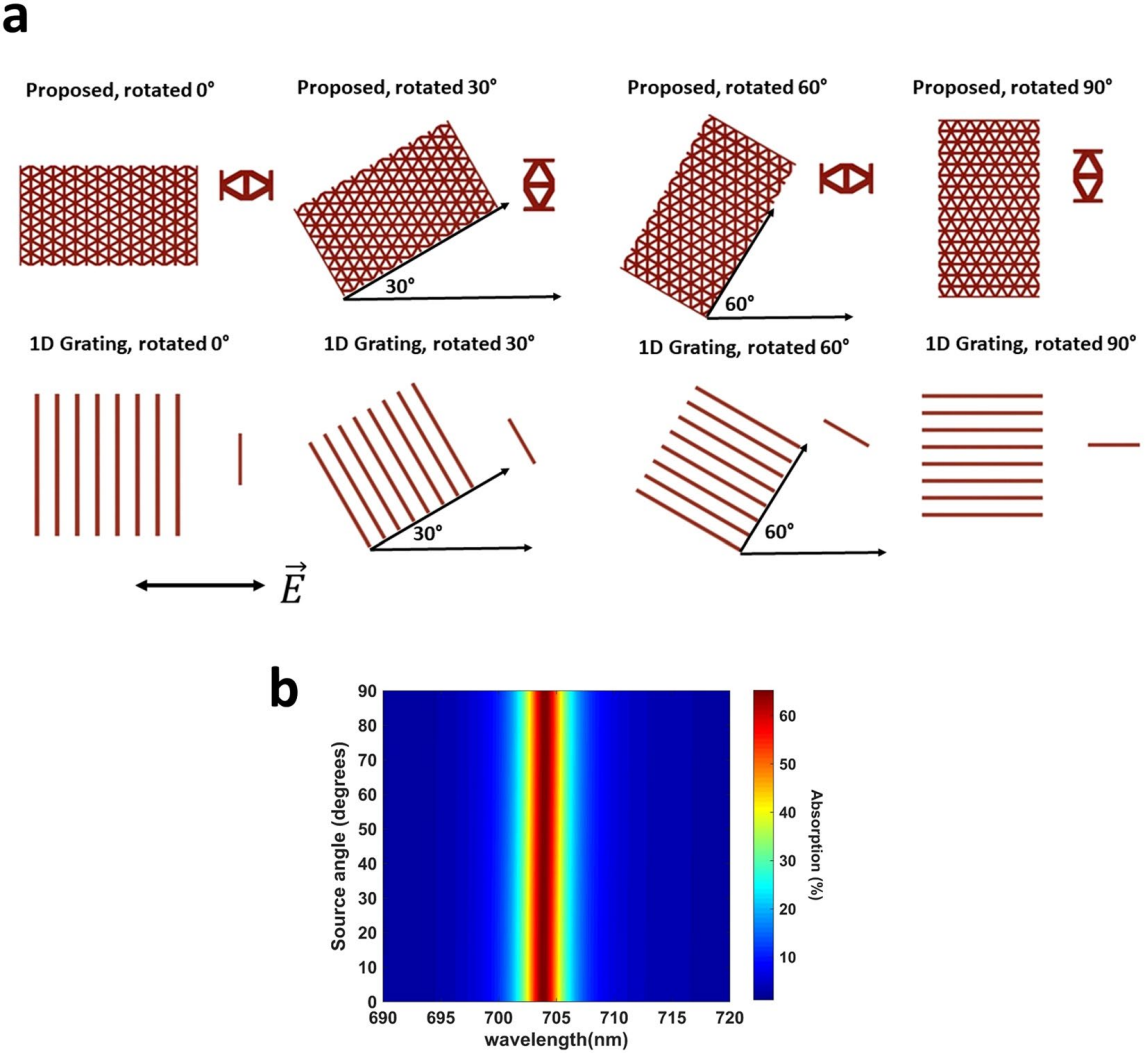
(**a**) Comparison of the unit-cells of the proposed design and the 1D-grating design under different rotations of Device I. (**b**) Absorption map of Device I for the polarization angles spanning from 0° (p, TM-polarization) to 90° (s, TE-polarization).

From Eqns 5 and 6, we see that it is the different interaction of different polarizations with the 1D grating that is causing polarization sensitivity. Thus, in an absolutely polarization insensitive design, the interaction of E-field with the unit-cell must be the same for all polarization angles. Equivalently, E-field with a fixed polarization angle has to “see” the same unit-cell upon rotating it arbitrarily^52^. To compare Device I with the devices using 1D grating, we kept the polarization angle at *ϕ* = 0 degrees, and rotated the unit-cells of each for 0, 30, 60, and 90 degrees. The result of this comparison is shown in Fig. 4a. From Fig. 4a, two main conclusions can be made. The first one is that although our grating cannot provide infinite rotational symmetry, its unit-cell repeats itself at every 60°, i.e. we have 60° of rotational symmetry. On the other hand, the 1D grating has different unit-cells, and hence different E-field-unit cell interactions for all polarization angles. Even though the proposed unit-cell for the 0° (60°) and 30° (90°) are different, the interaction of E-field with the unit-cells are somewhat identical. This is because, in the first case, E-field interacts perpendicularly with two gratings [one in the center, one in the corners (2 × 1/2)] and it is at 30 degrees of angle with the other four tilted ones. In the second case, however, E-field is parallel to the former two gratings, but it is at 60 degrees of angle with the tilted ones to compensate for the change in the former. In Fig. S2, we further demonstrate how different polarizations of light interact with the unit-cell. We explored the polarization independence of Device I in more detail by sweeping the polarization angle of the source from 0° to 90°. Our finding in Fig. 4b clearly shows that the structure maintains almost constant absorption at a nearly fixed spectral position.

### Technological tolerances, effect of geometrical parameters on GMRs

Results of the previous subsection demonstrate that GMRs can be excited when the phase-matching condition is satisfied, and the evanescent wave from the diffraction grating can be coupled to one of the guided waveguide modes. Although the waveguiding effects on the Bloch modes of the periodically patterned waveguide are complicated, we can still sufficiently understand how the spectral response of Device I depends on the structural parameters, by considering the optical field around graphene. Optical fields at the Ta_2_O_5_-PMMA (core-PMMA) and Ta_2_O_5_-SiO_2_ (core-substrate) boundaries are highly related to the effective mode index, n_eff_, of the guided-mode if we regard the structure as an asymmetric dielectric slab waveguide. This is done by considering the SWG as a medium with an effective dielectric permittivity^58^. Therefore, we start our discussion here by briefly reviewing the fundamental results of asymmetric dielectric slab waveguide theory^58,59,64^.

Eqs 7 and 8 are the guided-mode conditions in an asymmetric dielectric slab waveguide for TE and TM modes, respectively,

7 $$\tan (h{t}_{T{a}_{2}{O}_{5}})=\frac{p+q}{h(1-\frac{pq}{{h}^{2}})}$$

8 $$\tan (h{t}_{T{a}_{2}{O}_{5}})=\frac{\bar{p}+\bar{q}}{h(1-\frac{\bar{p}\bar{q}}{{h}^{2}})}$$

where $h={k}_{0}\sqrt{{n}_{T{a}_{2}{O}_{5}}^{2}-{n}_{eff}^{2}}$ is the transverse component of wavevector in the core, $p={k}_{0}\sqrt{{n}_{eff}^{2}-{n}_{SWG}^{2}}$ $q={k}_{0}\sqrt{{n}_{eff}^{2}-{n}_{Si{O}_{2}}^{2}}$ and are the decay constants in PMMA, SiO_2_ regions, respectively. *n*_*SWG*_ is the effective refractive index of the SWG, different for TE and TM modes. $\bar{p}$ and $\bar{q}$ are defined as $p\ast {n}_{T{a}_{2}{O}_{5}}^{2}/{n}_{SWG}^{2}$, $q\ast {n}_{T{a}_{2}{O}_{5}}^{2}/{n}_{Si{O}_{2}}^{2}$, respectively.

It is clear from Eqns 7 and 8 that n_eff_ is determined primarily by the thickness of the core to the wavelength ratio (normalized core thickness, ${{\rm{t}}}_{{{\rm{Ta}}}_{{\rm{2}}}{{\rm{O}}}_{{\rm{5}}}}$/λ) and the refractive indices of the core and cladding films. In Fig. S3 we show the solution of the transcendental mode-equations for our case, with varying fill-factor for the SWG. Significantly, the modes with higher n_eff_ are more tightly confined in the core, so they have low and almost equal leakage to both PMMA/graphene and substrate. However, for Device I, it is shown in Fig. 2b,c that most of the absorption is due to harnessing the reflection, and it is the transmission losses that prevent the absorption from reaching 100%. Since the transmission can never be made zero in a two-port system, the optimum design must have the maximum optical field at the core-PMMA boundary, while keeping that at the core-substrate boundary minimum. This would ensure that most of the leaked power of the guided-mode is exploited as absorption, and not transmission. In one extreme, if we increase n_eff_ to a value close to the core refractive index, the field confinement is better at the core, and so is the Q-factor of resonance. Therefore, the power leaking out to the graphene/PMMA becomes small and close to that leaking to glass. This results in nearly 50% absorption. In the other extreme, when n_eff_ is too small, the mode would leak too much energy to not only PMMA but also to substrate, so absorption again cannot dominate over transmission. Thus, obtaining maximum attainable absorption requires an intermediate n_eff_. Our discussion up to now highlights the fundamental trade-off and design consideration in the 2-port Device I, the changes to increase absorption and decrease the bandwidth also increase transmission to balance the former or even decrease it. To make things worse, increasing absorption would decrease the amplitude of the re-diffracted waves, so they cancel the originally diffracted waves less with destructive interference. This again increases transmission. Combining the phase-matching condition with this treatment of Device I, we will now explain the dependence of the absorption spectrum on geometrical parameters.

**Figure 5:**
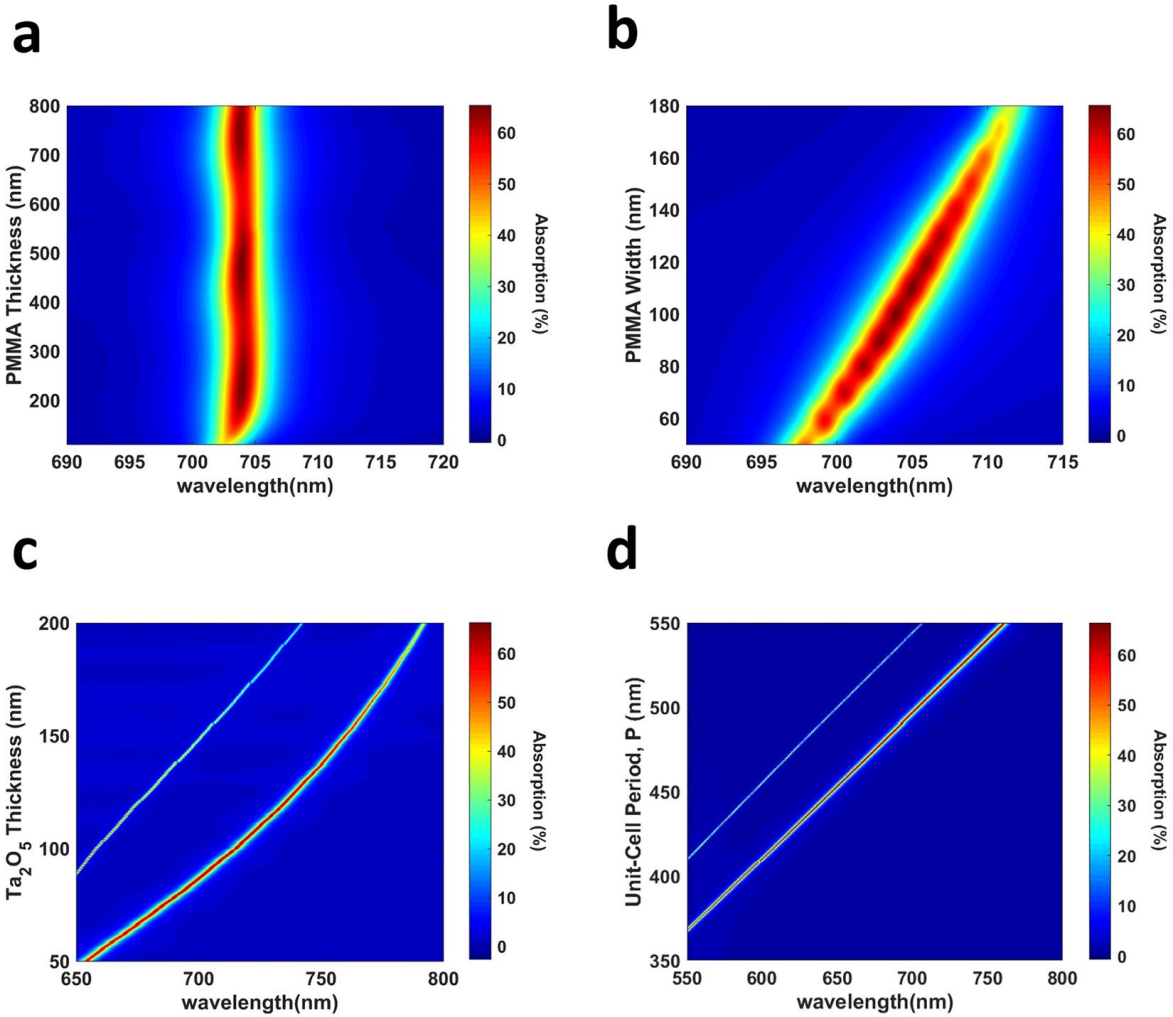
Dependence of absorption spectrum of Device I to structural parameters. Absorption spectrum of Device I under varying (**a**) PMMA thickness, t_PMMA_, (**b**) PMMA width, w, (**c**) Ta_2_O_5_ thickness, ${{\rm{t}}}_{{{\rm{Ta}}}_{{\rm{2}}}{{\rm{O}}}_{{\rm{5}}}}$, (**d**) unit cell periodicity in y-direction, P. When varying one parameter, we kept the other parameters at their optimum value. In the unit-cell periodicity sweeps, width of grating is kept as w = 0.2 P. The results are taken with p-polarized incident wave.

We first concentrate on the effect of PMMA thickness, t_PMMA_ on the absorption spectrum of Device I. For this purpose, we varied it over a very broad range, from 110 nm to 800 nm. The absorption spectrum in Fig. 5a shows that GMR excited at 703.82 nm is robust with respect to PMMA thickness. This implies that the PMMA thickness has only a tiny effect on the field configuration inside the core, agreeing well with the previously reported results^25,47^. There are two main points to be made from Fig. 5a. First of all, when the PMMA thickness is below a certain value, the resonance becomes weaker, because optical field around graphene decreases. This happens because the mode radiatively decays without being recombining with itself, as explained by the complex coupling coefficient between the incident field and guided-mode^36^. Secondly, the oscillation in the absorption spectrum starting after t_PMMA_ = 200 nm also agrees well with ref.^37^, and it arises because of the constructive and destructive interferences between the diffracted fields from the bottom and top of grating.

Next, we investigated the effect of the width of the grating, w. The value of w is changed from 50 nm to 180 nm and we obtained the absorption map in Fig. 5b. We observed that an intermediate value of PMMA SWG width is required for the maximum possible absorption, and the resonance wavelength red-shifts as the thickness increases. Both of these can be explained by considering the fill-factor of SWG that changes the effective refractive index of it^42,46,47,58,63^. Increasing PMMA width increases the fill-factor of the grating. Thus, the SWG assumes a larger effective refractive index for both polarizations, as outlined in Eqns 5 and 6 for the 1D-grating. Then, the effective mode index increases if we compare n_SWG_ = 1.3 and n_SWG_ = 1.4 cases in Fig. S3. Eq. 3 for 1D grating, therefore, requires the red-shifting of resonance wavelength. The same argument applies to our 2D grating as well. When w is too high, the absorption is small because there is a considerable amount of Fresnel reflection from the air-PMMA boundary that cannot be converted to absorption. Increased n_eff_ also decreases the leakage to PMMA/graphene. On the other hand, when w is too small, the effective mode index reduces too much toward the mode cut-off that the leakage to SiO_2_ starts dominating. The second explanation can be that the coupling efficiency is decreased with the thin grating. From this result, we observe that Device I can maintain the absorption strength for a large interval of the grating width.

In Fig. 5c, we show how the thickness of the Ta_2_O_5_ core region of the waveguide, ${{\rm{t}}}_{{{\rm{Ta}}}_{{\rm{2}}}{{\rm{O}}}_{{\rm{5}}}}$, effects the response of Device I. Core thickness has a strong influence of n_eff_, as shown in Eqns 7 and 8, so it also has a large impact on the spectral position of resonance under a fixed grating period, as Eq. 3 shows for the 1D grating. This concept qualitatively applies to our 2D grating as well. Figure 5c shows that the spectral position of the resonance changes almost linearly with Ta_2_O_5_ thickness, making the GMRs easily tunable, but also prone to fabrication inaccuracies. It is worth mentioning that the change in the resonance wavelength is smaller in ratio than that in the ${{\rm{t}}}_{{{\rm{Ta}}}_{{\rm{2}}}{{\rm{O}}}_{{\rm{5}}}}$ because the wavelength needs to simultaneously satisfy both the phase-matching condition and the mode-equation with constant grating period, while ${{\rm{t}}}_{{{\rm{Ta}}}_{{\rm{2}}}{{\rm{O}}}_{{\rm{5}}}}$ only controls the mode-equation^58,59,64^. For example, when ${{\rm{t}}}_{{{\rm{Ta}}}_{{\rm{2}}}{{\rm{O}}}_{{\rm{5}}}}$ is increased, the increase in the resonance wavelength is smaller in ratio, so ${{\rm{t}}}_{{{\rm{Ta}}}_{{\rm{2}}}{{\rm{O}}}_{{\rm{5}}}}$/λ ratio increases. This increases n_eff_, agreeing with Eq. 3. Finally, when ${{\rm{t}}}_{{{\rm{Ta}}}_{{\rm{2}}}{{\rm{O}}}_{{\rm{5}}}}$ is increased too much, n_eff_ increases too, so we see that the absorption decreases and stays near 50%. This supports our previous argument that under large confinement to the core, absorption and transmission approach to 50% due to low and almost equal leakage to both PMMA/graphene and substrate. In Fig. 5c, we also see the excitation of a second, narrower resonance that occurs at smaller wavelengths with weaker absorption strength. Hence, Device I can be used to simultaneously excite and tune the GMRs.

The last parameter that we modified is the periodicity, P, of the unit-cell. The absorption map shown in Fig. 5d indicates that resonance wavelength changes linearly with the unit-cell periodicity, but the effect on n_eff_ is opposite to that of ${{\rm{t}}}_{{{\rm{Ta}}}_{{\rm{2}}}{{\rm{O}}}_{{\rm{5}}}}$. To exemplify this, while increasing ${{\rm{t}}}_{{{\rm{Ta}}}_{{\rm{2}}}{{\rm{O}}}_{{\rm{5}}}}$ under fixed P increases n_eff_, increasing the periodicity under fixed ${{\rm{t}}}_{{{\rm{Ta}}}_{{\rm{2}}}{{\rm{O}}}_{{\rm{5}}}}$ increases the resonance wavelength by a smaller ratio to decrease n_eff_, from Eq. 3. This satisfies the mode-equations, too, because decreasing ${{\rm{t}}}_{{{\rm{Ta}}}_{{\rm{2}}}{{\rm{O}}}_{{\rm{5}}}}$/λ ratio also results in smaller n_eff_^58,59,64^. This is the main reason why Fig. 5d shows a low absorption strength, but smaller bandwidth when the period is small because the effective mode index is high. Lastly, the linear dependence of resonance position to the periodicity gives Device I great tunability, but also sensitivity to fabrication imprecisions.

### Achieving perfect absorption with ultra-narrow bandwidth

In a two-port system, such as Device I, perfect absorption can be achieved if the conditions of coherent perfect absorption (CPA) are satisfied, whereupon the interference and dissipation leads to perfect absorption^43,47,65^. However, the optical properties of graphene in the visible range are not suitable to construct a coherent perfect absorber^43,47^. In Device I, the main reason why 100% absorption cannot be achieved was the considerable amount of light that leaves the system, acting as transmission losses. In this respect, the total absorption of light can be achieved if the transmission channel is suppressed^25^. This can be accomplished by placing either a metallic mirror or a dielectric Bragg mirror^43,46^. Then, all of the previously transmitted power can be redirected back to graphene and interact with it. Coupled mode theory with time reversibility and energy conservation arguments result in the absorption spectrum described by the Lorentzian function in Eq. 9^66^.

9 $$A(\omega )=\frac{4\delta {\gamma }_{e}}{{(\omega -{\omega }_{0})}^{2}+{(\delta +{\gamma }_{e})}^{2}}$$

where *ω*_0_ is the resonance frequency, *γ*_*e*_ is the external leakage rate of the resonator and *δ* is the intrinsic loss rate due to monolayer graphene. Critical coupling and total absorption of incident light is achieved when the system is at resonance and when the leaked energy from the guided-mode is matched to the intrinsic absorption rate in graphene^25,43–46,58,67^.

**Figure 6:**
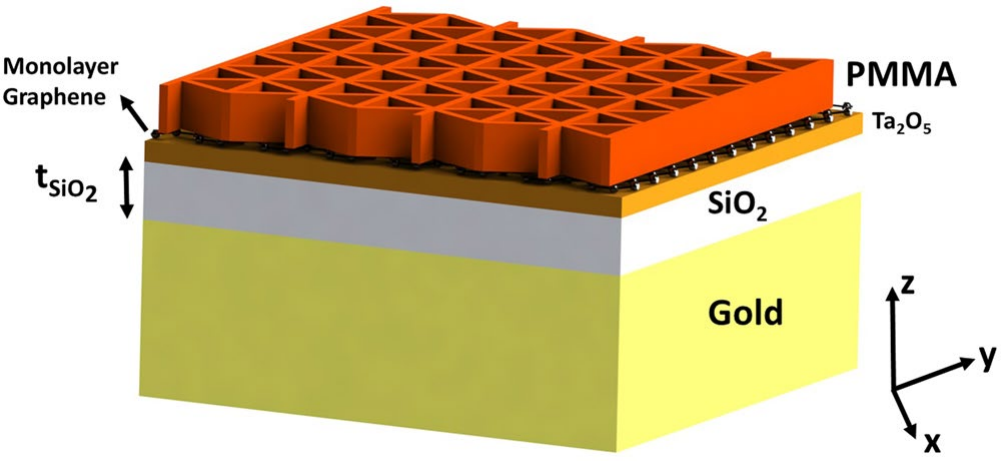
Structure of Device II, used to achieve perfect absorption by critically coupling graphene to excited GMRs. ${{\rm{t}}}_{{{\rm{SiO}}}_{2}}$ denotes the SiO_2_ thickness.

To achieve perfect absorption and retain the sub-micron thickness of Device I, we added a thick gold layer beneath Device I, to arrive at Device II, shown in Fig. 6. The gold layer in Device II acts a back-mirror. Its thickness, taken as 100 nm in simulations, is much larger than the skin-depth of the light at the wavelength range of interest, so the amount of light leaving Device II is insignificant. Two-port Device I, therefore, became one-port Device II. Gold layer of Device II can be replaced by a dielectric Bragg mirror, which would make the device all-dielectric and give a wider tunability range in the visible spectrum, at the expense of a more difficult fabrication process and bulkier design. Since there is negligible transmission, the absorption can now be found by simply *A* = 1 − *R* formula, and maximizing it requires minimizing the reflection, which is due to the externally leaked energy from the system that is not harnessed on graphene. This can occur due to either undercoupling or overcoupling of the GMR to graphene^43^. In both cases, the external leakage rate is not equal to the intrinsic absorption in graphene. The fact that critical coupling depends on the external leakage rate makes SiO_2_ thickness a critical parameter, as it controls the FP cavity modes in it^46^ and consequently reflection losses^25^.

**Figure 7:**
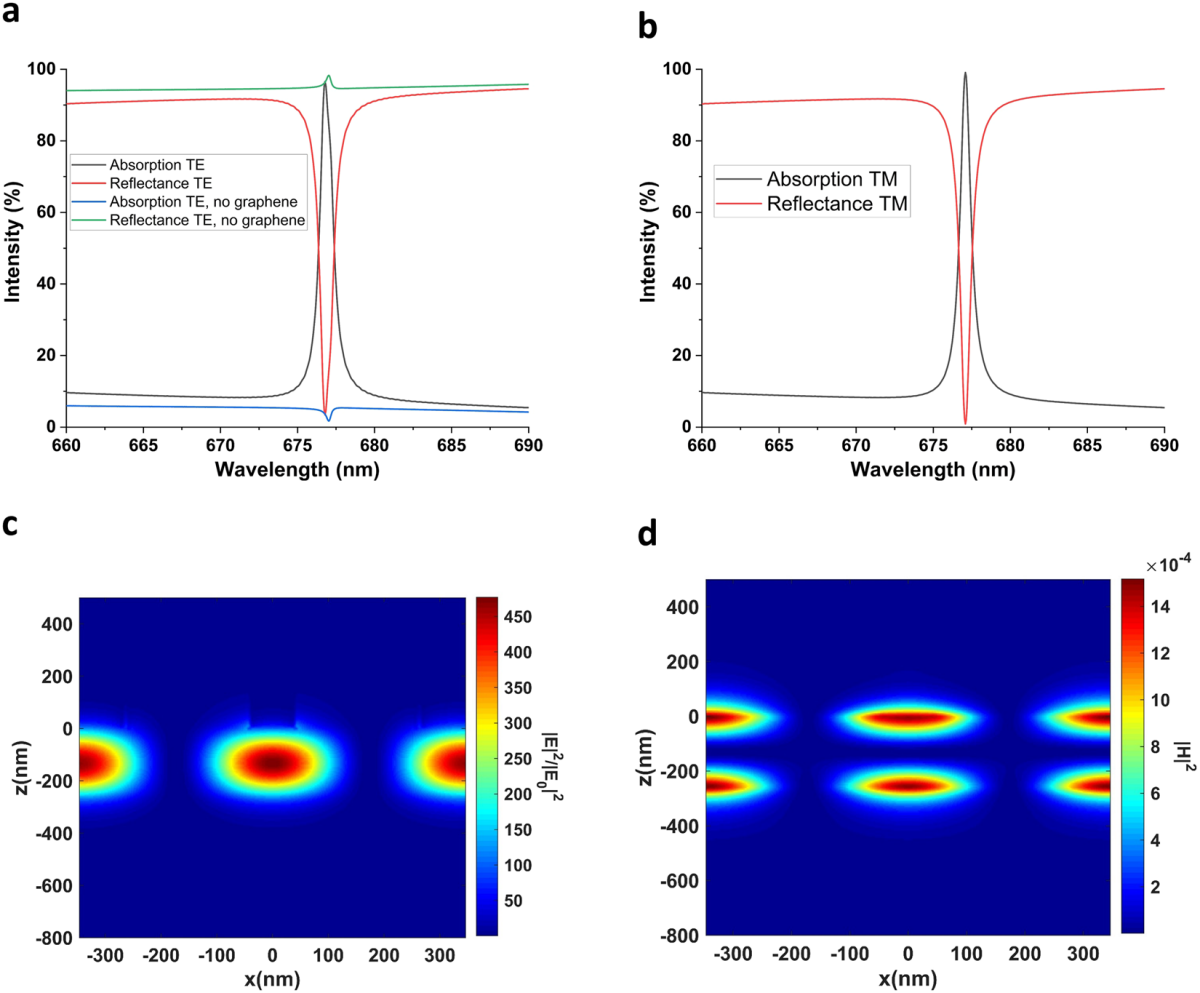
Achieving perfect absorption by critically coupling guided-mode to graphene. Absorption spectrum of Device II under (**a**) s-polarized, (**b**) p-polarized light. (**c**) |E|^2^/|E0|^2^ at XZ plane during resonance (**d**) |H|^2^ at XZ plane during resonance. (**a**) Also shows the absorption spectrum in the absence of monolayer graphene.

To achieve critical coupling, we set the geometrical parameters to their optimized values, i.e. ${{\rm{t}}}_{{{\rm{Ta}}}_{{\rm{2}}}{{\rm{O}}}_{{\rm{5}}}}$, P, w, t_PMMA_, and ${{\rm{t}}}_{{{\rm{SiO}}}_{2}}$ to 260 nm, 400 nm, 80 nm (0.2 P), 350 nm, and 360 nm, respectively. In the simulations, the dispersion of gold is modeled by ellipsometric measurements. The absorption spectrum for Device II with its optimized parameters is shown in Fig. 7a,b, under s and p-polarized light, respectively. In these figures, we see a very sharp resonance at 676.8 nm (677 nm) with the absorption strength of 98% (99.2%) for s (p)-polarized light. The bandwidth of the resonance for both TE and TM polarization is 0.8 nm, thus giving a very high Q-factor of about 850. Here, Q-factor is defined by Q = λ/Δλ. Figure 7a also demonstrates the absorption of Device II in the absence of graphene. The absorption without graphene is as low as 1%, because the layer thicknesses (including ${{\rm{t}}}_{{{\rm{SiO}}}_{2}}$) are arranged carefully so that complete reflection occurs at resonance. When present, graphene is then responsible for the remaining 98% absorption as well as a slight blue-shift of GMR. Figure 7c,d demonstrate the localization of electric and magnetic field in the core region of the waveguide. Comparing Figs 7c,d to 3a–c shows that Device II stores larger energy in the core region, and the Q-factor achieved in Device II is higher as a result.

We now want to emphasize the key design strategy in achieving the ultra-narrow bandwidth. To achieve critical coupling in a system where the external leakage rate is low, a very high energy storage, or equivalently, a very high-Q GMR in the resonant structure is needed^43,45^. Thus, the bandwidth is narrower in devices where graphene is placed far away from the optical field confined by GMR. Although this strategy results in a narrower bandwidth, the necessity of creating a very high-Q GMR puts vigorous stress on the fabrication process. To increase the Q-factor of resonances in Device II, it is better to confine the mode to the core, i.e. increase n_eff_. The narrower resonances at larger core thicknesses and smaller unit-cell periods in Fig. 7c,d supports this argument. Therefore, the principal strategy in controlling the Q-factor is the thickness of the core region, ${{\rm{t}}}_{{{\rm{Ta}}}_{{\rm{2}}}{{\rm{O}}}_{{\rm{5}}}}$, and the unit-cell periodicity, P. Increasing n_eff_ increases the energy storage inside the core region and decreases the leakage to the graphene^58^. The critical coupling condition is then satisfied by the very high-Q GMR, and the resultant bandwidth is low. This is the reason why ${{\rm{t}}}_{{{\rm{Ta}}}_{{\rm{2}}}{{\rm{O}}}_{{\rm{5}}}}$ in Device II is much higher than that of Device I, and P in Device II is smaller than that in Device I. t_PMMA_ and w are tailored accordingly to meet critical coupling condition.

**Figure 8:**
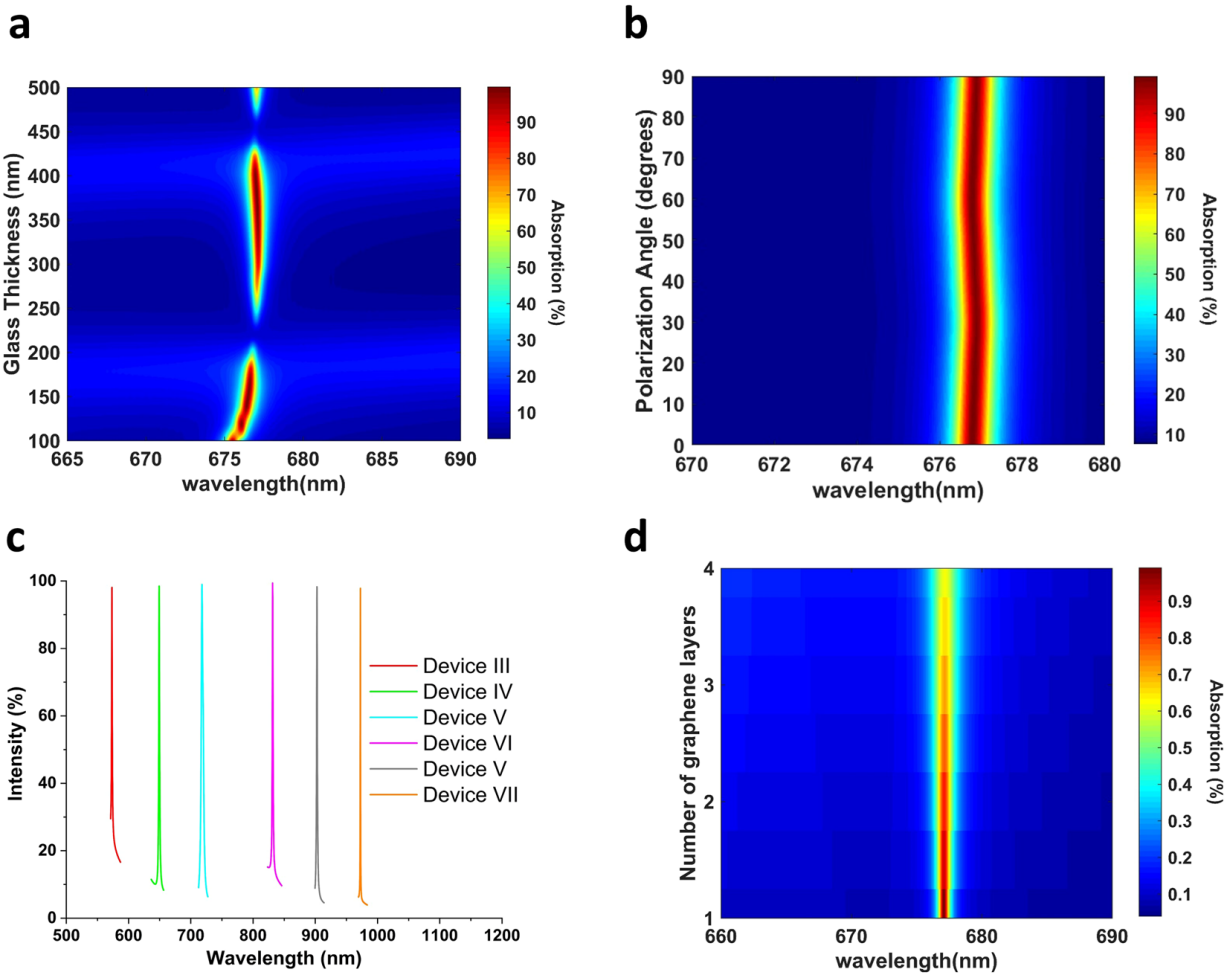
Absorption spectrum of Device II under varying **(a)** Glass thickness, ${{\rm{t}}}_{{{\rm{SiO}}}_{2}}$, (**b**) Polarization angle. (**c**) Tuning the perfect absorption in the visible and mid-infrared ranges. ${{\rm{t}}}_{{{\rm{Ta}}}_{{\rm{2}}}{{\rm{O}}}_{{\rm{5}}}}$ = 160, 180, 300, 300, 400 nm; P = 350, 400, 550, 500, 600 nm, ${{\rm{t}}}_{{{\rm{SiO}}}_{2}}$ = 380, 420, 260, 240, 540 nm for Devices 3, 4, 5, 6 and 7, respectively. t_PMMA_ = 350 nm and w = 0.2 P, for all designs. (**d**) Layer-dependent absorption of Device II.

To examine the effect of the SiO_2_ thickness in the absorption spectrum, we varied it over a wide range from 100 nm to 500 nm, and the result is shown in Fig. 8a. The key observation is that the absorption spectrum shows maxima and minima that are periodic with λ/${{\rm{2n}}}_{{{\rm{SiO}}}_{2}}$, λ being the resonance wavelength. This is consistent with the results reported in refs^25,46^ and it is because of the phase-shift introduced by the SiO_2_ layer and the FP modes defined by it^46^. Another reason may be that the system evolves from critical coupling to overcoupling or undercoupling, as altering glass thickness changes external leakage rate^43^, while the intrinsic loss rate remains almost the same. An interesting observation is that glass thickness starts having an effect on resonance wavelength below a certain value. Besides this, when ${{\rm{t}}}_{{{\rm{SiO}}}_{2}}$ is close to the value resulting in absorption maxima, an increase in it causes the bandwidth to increase. This is because the external leakage rate controls the bandwidth of absorption, described by Eq. 9.

We inspected the polarization-independence of Device II, and the result is shown in Fig. 8b. In terms of the absorption strength, Device II shows slightly more polarization dependence than Device I. This is because, as the Q-factor of GMR is increased, meeting the critical coupling condition becomes stricter. Hence, the spectrum becomes more sensitive to the energy stored in the GMR. Slight variations in this energy can then disturb the critical coupling condition. Nevertheless, the absorption strength is still above 98% for all source angles, and the spectral position of resonance shows even less dependence on polarization. It is worthwhile to mention that the proposed structure cannot become polarization insensitive if the thin absorbing film is inherently anisotropic. An example for such a case would be phosphorene. In that case, the puckered configuration of phosphorene and the resulting anisotropy^68^ means that although the grating has 60° rotational symmetry, overall structure will not have this feature.

The sensitivity of GMR to geometrical parameters gives Device II a great tunability in visible and NIR ranges. As a general guideline, the resonance can be red-shifted by an increase in ${{\rm{t}}}_{{{\rm{Ta}}}_{{\rm{2}}}{{\rm{O}}}_{{\rm{5}}}}$ and P, as in Fig. 7c,d. To decrease the bandwidth, on the other hand, it is necessary to increase ${{\rm{t}}}_{{{\rm{Ta}}}_{{\rm{2}}}{{\rm{O}}}_{{\rm{5}}}}$ but decrease P, so that n_eff_ increases. ${{\rm{t}}}_{{{\rm{SiO}}}_{2}}$ is picked accordingly to satisfy the critical coupling condition. Tuning of the GMR between 570 nm and 970 nm is shown in Fig. 8c. Device V in Fig. 8c is also an example of achieving multiple resonances from a single device. Device VII has an FWHM of only 0.5 nm, reaching an ultra-high Q-factor of approx. 2000. This is because graphene’s intrinsic absorption starts declining for wavelengths larger than 850 nm. The main drawback of our design in terms of tunability is that the intrinsic absorption of gold starts to be significant below ~600 nm. Therefore, although 100% absorption can be achieved below 550 nm easily, it is not graphene that is responsible for perfect absorption. Replacing the gold mirror with dielectric Bragg mirror can extend the perfect absorption in graphene in the visible range. Aluminum and silver can also be used as the back-mirror, as their absorption starts dominating at significantly smaller wavelengths, but the latter’s degradation in air is an issue to consider in fabrication process^69^.

We now accentuate the fundamental difference in the design methodologies of Device I and Device II. In Device I, the effective mode refractive index had to be kept at an intermediate value because too high or too weak confinement of the mode to the core was detrimental to absorption. However, in Device II we could be able to confine the mode as much as we can to the core, without considering transmitted losses, and critical coupling gives both perfect absorption and ultra-narrow bandwidth. The same design strategy in Device I would result in absorption and transmission approaching each other, and to 50%.

Up to now, this paper dealt with achieving perfect absorption with monolayer graphene. However, the concept of critical coupling is general and can be applied to other 2D materials, such as transition-metal dichalcogenides (TMDs), and multilayer graphene to yield perfect absorption with polarization insensitivity. To explain the effect of multilayer graphene, we increased the number of graphene layers in the simulations, and the result is demonstrated in Fig. 8d. The result of the addition of graphene layers is the decrease in absorption strength and the broadening of the absorption peak, without a change of resonance wavelength. All of these results are well-expected from coupled mode theory and Eq. 9. Increasing the number of graphene layers would not affect the external leakage rate of the resonator as it is controlled by the mode confinement to the core. However, the intrinsic absorption rate would increase and the match between the external leakage rate and intrinsic absorption rate would then be lost. So, increasing the graphene layers drives the resonator towards undercoupling^66^. Other 2D materials, such as transition-metal dichalcogenides show stronger light-matter interaction than graphene, in the order of 10% in the visible range^70^. Compared to the intrinsic 2.3% absorption in graphene, larger intrinsic absorption in TMDs would mean satisfying the critical coupling condition requires a larger external leakage rate for matching. So, the absorption spectrum associated with them are inherently broader. From the viewpoint of light sensing or biosensing applications, using graphene is advantageous due to narrower bandwidth. However, the broader resonances associated with TMDs would mean a larger average absorption in the visible spectrum and the presence of a direct bandgap combined with it makes monolayer TMDs a great candidate for photodetection and photovoltaics^71,72^.

The experimental verification of this work starts with the fabrication flow of choosing a Silicon or quartz substrate. Choice of substrate does not affect Device II as the thick gold layer means that there will be insignificant amount of light reaching there. Thus, a thin (5–10 nm) Titanium layer can be coated beneath gold for better adhesion, without affecting the optical properties. After that gold, SiO_2_ and Ta_2_O_5_ can all be coated by Physical Vapor Deposition (PVD) methods such as sputtering. Chemical Vapor Deposition (CVD) grown graphene can be transferred and PMMA grating structure can be realized by electron-beam lithography. The numerical findings of this paper propose that the core thickness and the unit-cell periodicity are the most important factors in determining the resonance position and bandwidth, as well as the absorption strength. This makes the precise deposition of Ta_2_O_5_ and electron-beam lithography of the 2D grating structure the most important steps. The fabricated samples can be characterized by Fourier Transform Infrared Spectroscopy (FTIR) technique for the resonances in NIR, whereas confocal microscopy can be utilized for resonances in visible range. We believe that the deviations of experimental findings from theoretical results can be restricted to a controlled region by the physical laws and explanations presented in this paper.

## Conclusions

In conclusion, the enhancement of light-graphene interaction is numerically analyzed in this paper and the near-perfect polarization insensitivity of proposed design to incident light is verified. Our novel sub-wavelength dielectric grating excites the guided modes of an asymmetric dielectric slab waveguide. The critical coupling of the guided mode resonances to graphene resulted in perfect light absorption that is tunable by structural parameters in the visible and NIR ranges. The bandwidth of the resonance, 0.8 nm resulted in a very high Q-factor of 850. To the best of our knowledge, this bandwidth is smaller than all of the relevant device proposals with sub-micron thickness. Overall, our simple yet robust, and easy-to-fabricate device holds great promise in ultra-sensitive color detection and design of advanced photodetectors. Our future work will focus on the fabrication of the device and extending the tunability range of the guided-mode resonance.

## Electronic supplementary material

Supplementary Information
