# Publisher Correction: Near-absolute polarization insensitivity in graphene based ultra-narrowband perfect visible light absorber

**DOI:** 10.1038/s41598-018-36221-6

**Published:** 2018-11-30

**Authors:** Deniz Umut Yildirim, Amir Ghobadi, Ekmel Ozbay

**Affiliations:** 1 NANOTAM-Nanotechnology Research Center, Bilkent University, 06800 Ankara, Turkey; 2 Department of Electrical and Electronics Engineering, Bilkent University, 06800 Ankara, Turkey; 3 Department of Physics, Bilkent University, 06800 Ankara, Turkey; 4 UNAM-Institute of Materials Science and Nanotechnology, Bilkent University, Ankara, Turkey

Correction to: *Scientific Reports* 10.1038/s41598-018-33609-2, published online 12 October 2018

The original version of this Article contained an error in the title of the paper, where the word “graphene” was incorrectly given as “grapheme”. This has now been corrected in the PDF and HTML versions of the Article.

