# Supplementary material for: Near-absolute polarization insensitivity in graphene based ultra-narrowband perfect visible light absorber
Source: Sci Rep. 2018 Oct 12;8:15210. doi: 10.1038/s41598-018-33609-2 (PMC6185963; doi:10.1038/s41598-018-33609-2)
Supplement: Supplementary file 1 — Supplementary Information [file 41598_2018_33609_MOESM1_ESM.pdf]

Supplementary Information

Near-absolute polarization insensitivity in graphene based ultra-narrowband perfect visible light absorber

Deniz U. Yildirim,[*,†,‡] Amir Ghobadi,[†,‡] and Ekmel Ozbay[*,†,‡,¶,§]

†*NANOTAM-Nanotechnology Research Center, Bilkent University, 06800, Ankara, Turkey.*

‡*Department of Electrical and Electronics Engineering, Bilkent University, 06800, Ankara, Turkey.*

¶*Department of Physics, Bilkent University, 06800, Ankara, Turkey.*

§*UNAM-Institute of Materials Science and Nanotechnology, Bilkent University, Ankara, Turkey.*

E-mail: yildirim@ee.bilkent.edu.tr; ozbay@bilkent.edu.tr

The equivalent unit-cell of our sub-wavelength grating is shown in Fig.S1. It is essentially an equilateral hexagon with a side length of P.

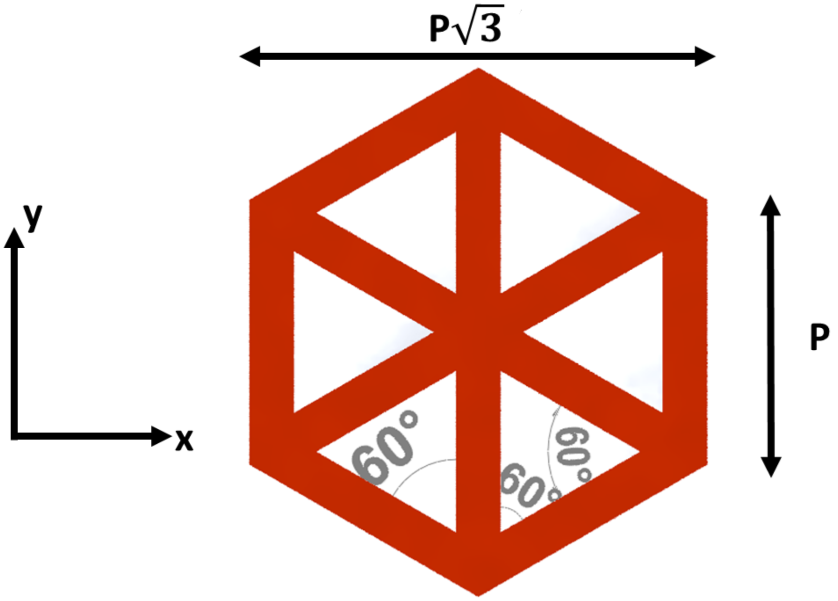

Figure S1: Equivalent unit-cells for Devices I,II,III,IV,V,VI,VII; detailing the sub-wavelength grating

(a)

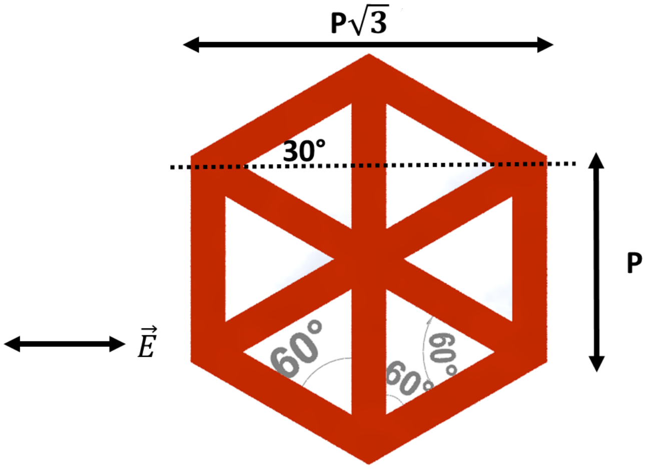

(b)

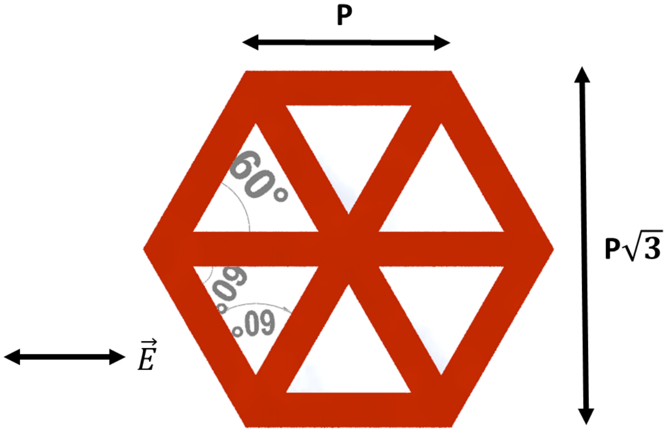

Figure S2: The interaction of the p-polarized incident wave with the unit-cell, rotated **(a)** 0° and **(b)** 90°.

. . . .

It is maybe more obvious compared to the unit-cell presented in the the main text that the structure has 60° rotational symmetry. In Fig.S2, we show the interaction of incident light, polarized in x-direction (p-polarized,TM) with the same unit-cell, rotated 0° and 90°.

When the unit-cell is rotated 0° as in Fig.S2a, the incident light is perpendicular to the vertical grating ridges. We call these Grating Group 1. Among the Grating Group 1, the middle ridge is twice the length of the side ones. Thus, E-field has four (1+1+2)

perpendicular incidences to these ridges. In addition, E-field is at 30° to the titled ridges, which we call Grating Group 2. Since there are 2*(1+1+2)=8 titled ridges, $8 * sin(30°) = 4$ perpendicular incidences are added. There are $8 * sin(40°) = 4\sqrt{3} \approx 7$ parallel incidences to the ridges.

On the other hand, when the unit-cell is rotated 60° as in Fig.S2b, the incident light is now parallel to the Grating Group 1. So, E-field has four parallel incidences to these ridges. Also, E-field is now at 60° to the Grating Group 2, so $8 * sin(30°) = 4$ perpendicular incidences are added. There are $8 * sin(60°) = 4\sqrt{3} \approx 7$ perpendicular incidences to the ridges. Thus, the Grating Group 2 compensate for the decrease in the perpendicular incidences to the Grating Group 2.

Another reason for adopting this unit-cell is that the distance between all of the parallel ridges are the same, i.e. $P\sqrt{3}/2$, adding further to the symmetry of the unit-cell.

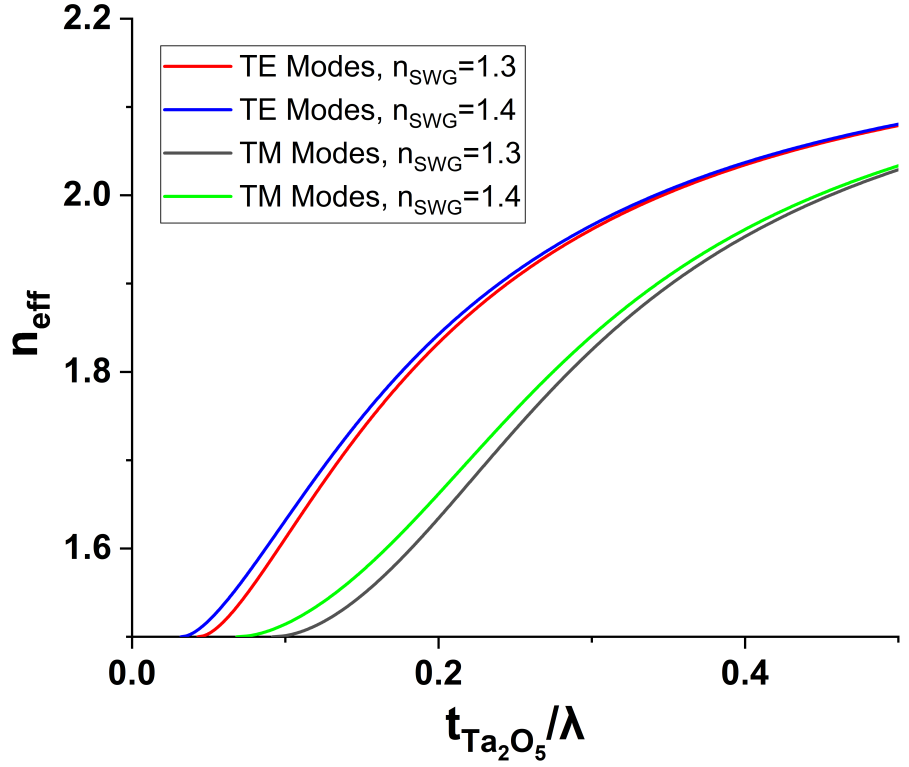

Figure S3: Effective refractive index versus the normalized core thickness for an asymmetric dielectric slab waveguide for different fill factors, or effective refractive indices, of SWG. Refractive indices for the core and substrate are taken as $n_{Ta_2O_5}$=2.2 and n_{SiO_2}=1.5, respectively.

Finally, we show the solution of the transcendental waveguide equation for TE and TM modes in an asymmetric dielectric slab waveguide in Fig.S3. As the filling ratio of the grating is increased, so is its effective refractive index. It can be observed from Fig.S3 that this also increases the effective mode index for both TE and TM modes.
